# Overexpression p21WAF1/CIP1 in suppressing retinal pigment epithelial cells and progression of proliferative vitreoretinopathy via inhibition CDK2 and cyclin E

**DOI:** 10.1186/1471-2415-14-144

**Published:** 2014-11-25

**Authors:** Ying Wang, Zhigang Yuan, Caiyun You, Jindong Han, Haiyan Li, Zhuhong Zhang, Hua Yan

**Affiliations:** Department of Ophthalmology, Tianjin Medical University General Hospital, Tianjin, 300052 China; Shanxi Eye Hospital, Taiyuan City, Shanxi Province 030002 China

**Keywords:** P21^Waf1/Cip1^, Cell cycle arrest, Retinal pigment epithelial cells, Proliferative vitreoretinopathy

## Abstract

**Background:**

P21 is one kind of cyclin-dependent kinase inhibitor that can prevent cells from going through the G1/S phase checkpoint and inhibit cell proliferation. Proliferative vitreoretinopathy (PVR) is a proliferative response in the eye. The aim of this study was to determine whether p21^Waf1/Cip1^ (p21) suppresses the proliferation and migration of retinal pigment epithelial (RPE) cells *in vitro* and controls PVR development *in vivo*.

**Methods:**

Cell cycle analyses and transwell assays were conducted to assess cell proliferation characteristics and the migration ability of RPE cells after transfection with *p21*. Western blot and reverse-transcription polymerase chain reaction technologies were used to detect the expression of p21, CDK_2_ and cyclinE in RPE cells and rabbit retinal tissues. The impact of increasing p21 expression on PVR development was conducted by implantation of an adenovirus vector containing rabbit *p21* (rAd-p21) in a PVR rabbit model. The prevalence of PVR and retinal detachment was determined by indirect ophthalmoscopy on days 3, 7, 14, and 21 after the injection of rAd-p21 into the vitreous. B scans and hematoxylin-eosin staining were employed to check rabbit retinas on day 21.

**Results:**

Cell cycle analyses and transwell assays showed that p21 inhibited the proliferation and migration of RPE cells. Increased expression of p21 was detected in cultured RPE cells and rabbit retinas after transfection with the *p21* gene, whereas levels of CDK_2_ and cyclinE were decreased. The increase in p21 expression effectively suppressed the development of PVR in a rabbit model.

**Conclusions:**

The increase in p21 expression in RPE cells not only inhibits the proliferation and migration of RPE cells *in vitro*, but also suppresses the development of PVR *in vivo*, which indicates its therapeutic potential in treating PVR.

## Background

Proliferative vitreoretinopathy (PVR) is characterized by the migration and proliferation of cells following a break in the retina or trauma, leading to formation of periretinal membranes, followed by contraction of cellular membranes and traction on the retina that causes retinal detachment. The retinal pigment epithelium (RPE) is a critical cell layer that undergoes proliferation in PVR. In PVR, the RPE cells come into the vitreous and migrate to the vitreoretinal interface, where they proliferate and form traction epiretinal membranes. Effective treatment of PVR remains as a clinical challenge in ophthalmology. Although modern vitreoretinal microsurgery has obtained satisfactory results in the treatment of PVR, operational complications and/or poor visual acuity have been associated outcomes in many cases [1, 2]. Therefore, it is important to obtain a better understanding of the pathogenesis of this disease and to find new strategies for the prevention and treatment of PVR.

The p21 protein belongs to the family of cyclin-dependent kinase inhibitors (CDKIs), which play an important role in the regulation of cell cycle progression [3]. In cells, p21 forms quaternary complexes with cyclins, CDKs, and proliferating cell nuclear Ag, which are essential for cell cycle progression. Up-regulation of p21 expression and other CDKIs results in activation of suppression molecules of CDKs and cyclinE, allowing accumulation of hypophosphorylated Rb and cell cycle arrest in the G1 phase [4, 5]. On the contrary, decreased expression of p21 is associated with several proliferative diseases in the eye, such as pterygium and retinoblastoma [6, 7]. A previous study has shown that an increase in p21 by subconjunctival injection of the recombinant adenovirus vector-mediated p21 gene inhibits fibroproliferation and wound healing in a rabbit model of glaucoma filtration surgery [8].

Previous studies have suggested a role for p21 in tumorigenesis [9, 10]; however, its function in PVR has not been reported. In this study, we characterized the function of p21 in the proliferation and migration of RPE cells, and explored its possible role in experimental PVR. Our findings indicated that p21 might play a protective role in PVR and may be a new target for PVR treatment.

## Methods

### Reagents

The recombinant adenovirus vector-mediated *p21* gene (rAd-p21) and recombinant adenovirus empty vector (rAd-NC) were obtained from GeneChem Co. Ltd. (Shanghai, China). Human RPE cells (D407 cell line) were purchased from Chuan Xiang Biological Technology Co. Ltd (Shanghai, China). Platelet rich plasma (PRP) was isolated from the peripheral blood of healthy people. The study was approved by Tianjin Medical University General Hospital Medical Ethics Committee (201315). Written informed consent for blood sampling was obtained from all participants according to the Declaration of Helsinki. After centrifuging for 10 min (1500 rpm/min at 4°C), 25 ml of PRP containing platelets of 4.00 × 10^8^/ml was obtained.

### Cell culture and recombinant adenovirus vector-mediated *p21*gene transfection

Human RPE cells (D407 cell line) were cultured in 1640 medium with 10% fetal bovine serum (FBS; Hyclone, USA), 100 U/mL penicillin, and 100 μg/mL streptomycin at 37°C in an atmosphere of 5% CO_2_. Before transfection, cells were starved in serum-free medium for 24 hours. Quiescent cells were plated in six-well plates and divided into a phosphate-balanced solution (PBS) group, rAd-p21 transfection group and negative control group (rAd-NC group), and then were incubated overnight with PBS, rAd-p21 and rAd-NC, respectively. After 24 hours, the cells were washed and cultured with complete medium.

### Cell cycle analysis by flow cytometry

Human RPE cells (1 × 10^6^) in six-well plates were incubated with PBS, rAd-p21 and rAd-NC transfection reagents for 24 hours. The cells were harvested and fixed in 70% ethanol. Before analysis, cells were washed with PBS and resuspended in PBS (pH = 7.4). RNase (100 μg/ml) and propidium iodide (50 μg/ml) were added to suspend the cells for 30 min. The use of a Becton-Dickenson FACSVantage flow cytometer system (Becton-Dickenson, USA) determined the DNA histograms, and Cell Quest software version 3.2 (Becton-Dickenson, USA) was used to analyze the cell cycle distribution. The experiments were performed in triplicate and repeated three times.

### Transwell assay

Corning Transwell 3422 Chambers (Corning, USA) were used to assess human RPE cell migration in response to the *p21* gene. After 24 hours transfection, each group of cells (incubated with PBS, rAd-p21 or rAd-NC) was harvested and reseeded at a density of 2.5 × 10^4^ per well in the upper chamber with 8 μm membranes in serum-free 1640 medium. The 1640 medium containing 10% FBS was placed in the lower chamber. After 6 hours, non-migrating RPE cells were gently scrubbed and removed from the surface. Migrating cells attached to the membrane were fixed with 1% glutaraldehyde and stained with 0.1% crystal violet. To assess the average number of migrating cells, cells were counted in five random high power fields. All experiments were repeated three times.

### Western blot analysis

The cold radio-immunoprecipitation assay (RIPA) buffer and phenylmethylsulfonyl fluoride (PMSF; a protease inhibitor) were used to prepare the cells, which were washed twice with ice-cold PBS. A cell scraper was used to gather cell lysates, which were collected and transferred to a microcentrifuge tube and centrifuged for 15 minutes at 12,000 × g (4°C). The supernatant was collected and a BCA protein assay kit (Pierce, Rockford, USA) was used to measure the protein content of each lysate. Electrophoresis was performed (25 ug protein/well) with the use of a sodium dodecyl sulfate polyacrylamide gel electrophoresis gel, which was then transferred to a polyvinylidene difluoride membrane and analyzed by immunoblotting. The primary anti-p21 polyclonal antibody (1:200; Santa Cruz, California, USA), CDK_2_ polyclonal antibody (1:500; Santa Cruz, California, USA), cyclinE polyclonal antibody (1:500; Santa Cruz, California, USA) and antibody against β-actin (1:2000; Millipore, Massachusetts, USA) were used to immunodetect p21, CDK_2_, cyclinE and β-actin, respectively. Band densities of the p21, CDK_2_ and cyclinE proteins were all normalized to β-actin and quantified by ImageJ software. Western blot analyses were repeated three times.

### RNA isolation and reverse transcription polymerase chain reaction

According to the manufacturer’s instructions, TRIzol reagent (Invitrogen, Carlsbad, CA) was used to isolate total RNA from the RPE. Samples of RNA (about 1 μg to 2 μg of total RNA) were reverse-transcribed to cDNA with gene-specific primers using the TransScript RT-PCR System kit (Transgen, Beijing, China). The target gene single-stranded cDNA was amplified in a polymerase chain reaction (PCR), with sequence-specific primers of *p21* (forward primer: 5′-CCT TGT CCT TTC CCT TCA-3′, reverse primer: 5′-TCC TTG TTC CGC TGC TAA-3′, 355 bp), of *CDK2* (forward primer: 5′-ATC CGC CTG GAC ACT GAG-3′; reverse primer: 5′-GTG GAG GAC CCG ATG AGA-3′; 273 bp), of *cyclinE* (forward primer: 5′-TTC CAC ACA GGA GCA AAG TAT G-3′; reverse primer: 5′-TGC AAC TTT GGA GGG TAG ATT T-3′; 377 bp), and of *β-actin* (forward primer: 5′-AGT TGC GTT ACA CCC TTT C-3′; reverse primer: 5′-CAC CTT CAC CGT TCC AGT-3′, 147 bp). Expression of *β-actin* was used as the internal standard. Amplification was performed in a thermal cycler at 94°C for 30 s, at 56°C for 30 s, and at 72°C for 30 s over 35 cycles. Reverse-transcription (RT)-PCR products were electrophoresed on a 1.5% agarose gel and visualized with ethidium bromide. The intensities of the amplified cDNA fragments were estimated with a video-densitometer.

### Animals and induction of PVR

Thirty adult pigmented rabbits (2.0–2.5 kg) were purchased from the Beijing Shahe Tongli Experimental Animal Farm (Beijing, China). All animal experiments conformed to the protocols approved by the Animal Care and Use Committee of Tianjin Medical University (TMUaMEC2013008). All of the rabbits were anesthetized with an intramuscular injection of ketamine (50 mg/kg) and promethazine (25 mg/kg). Before the surgery, rabbit pupils were dilated with tropicamide and the rabbits were given antibiotics. PVR was induced by intravitreal injection of 2.5 × 10^5^ human RPE cells in 0.1 mL PRP with a 30-gauge needle. An anterior chamber paracentesis was made and approximately 0.1 mL aqueous humor was drained with a 30-gauge needle before the injection.

### Intravitreal injection of rAd-p21

All the rabbits were randomly divided into 3 groups randomly 7 days after PVR was induced: PBS group (*n* = 10, intravitreal injection of 3 μL PBS); empty vector group (*n* = 10, intravitreal injection of 3 μL rAd-NC); and gene treated group (*n* = 10, intravitreal injection of 3 μL rAd-p21). Guided by an operating microscope, rAd-p21, rAd-NC or PBS was injected into the superotemporal area posterior to the lens using a metal needle connected to a glass syringe (Hamilton Co., Reno, NV), taking care to avoid penetration of the lens or damage to the vortex veins. The injection site was 3 mm posterior to the superotemporal limbus. The injection site was visualized with a standard indirect ophthalmoscope and a 90-Dcondensing lens (Volk Optical Inc., Mentor, OH) after each injection. The fundus status of each rabbit was acquired on days 3, 7, 14 and 21. The findings were grouped into six stages according to Fastenberg et al. [11]; i.e., stage 0, no lesions; stage 1, intravitreal membranes; stage 2, retinal focal traction; stage 3, focal retinal detachment involving less than two quadrants; stage 4, extensive retinal detachment involving more than two quadrants; and stage 5, total retinal detachment, folds, and holes. All rabbits were anesthetized and examined with a B scan to access PVR and retinal detachment, and then they were sacrificed at the end of the experiments by anesthetic overdose.

### Hematoxylin-eosin staining

The eyes were enucleated and imbedded in 10% formalin for 2 days, and then imbedded in paraffin blocks, which were then cut into 5 μm thick slices and stained with hematoxylin-eosin (H&E). The slices were photographed with a digital camera after dehydration and fixation.

### Western blot analysis and reverse transcription polymerase chain reaction *in vivo*

The eyes were enucleated and the retinas were dissected and isolated from the RPE. The retinas were analyzed by western blot and were lysed directly on ice in 300 uL RIPA buffer and PMSF. The lysed retinas were centrifuged and the supernatants were transferred to new tubes, and then the supernatants were processed as described previously. The RT-PCR analysis of retinas was performed as described previously.

### Statistics

The data were analyzed with a one way analysis of variance and χ2 analysis. A value of p < 0.05 was considered to be statistically significant.

## Results

### Overexpression of p21 inhibits the cell cycle in human PRE cells

**Figure 1:**
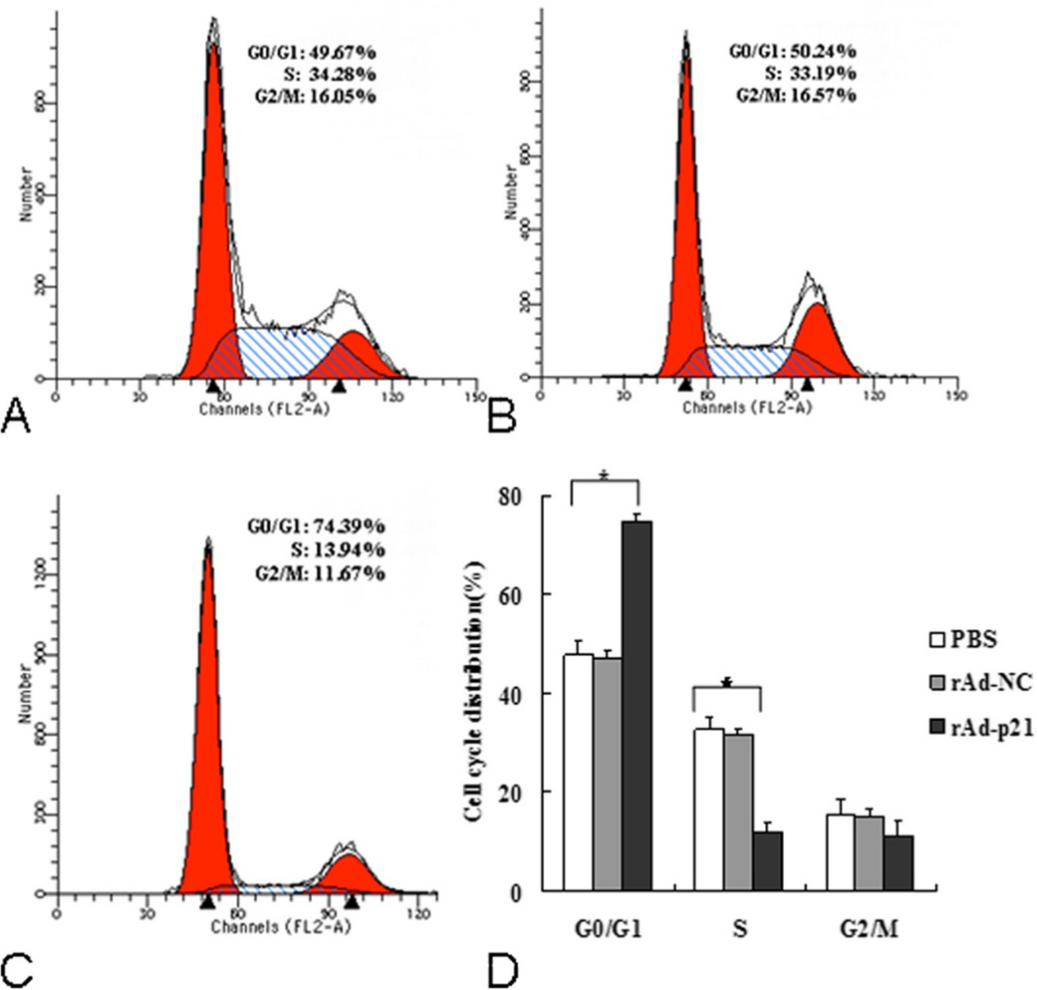
**Effect of the** ***p21*** **gene on the cell cycle of cultured human RPE cells. A**: RPE PBS, **B**: RPE rAd-NC, **C**: RPE rAd-p21, **D**: Bar graph represents the quantitative data of RPE cell cycle distribution in PBS, rAd-NC and rAd-p21 groups. Each value represents the mean ± SD of three independent experiments, each performed in triplicate (*P < 0.05).

We overexpressed p21 by transfection of rAd-p21. Subsequently, to determine whether p21 inhibits the proliferation of RPE cells, we performed flow cytometry to analyze the cell cycle progression. As shown in Figure 1, rAd-p21 induced G0/G1 phase cell cycle arrest compared with the PBS and rAd-NC groups. This result suggested that up-regulation of p21 could inhibit the proliferation of human RPE cells.

### Effect of the *p21*gene on the migration of human RPE cells

**Figure 2:**
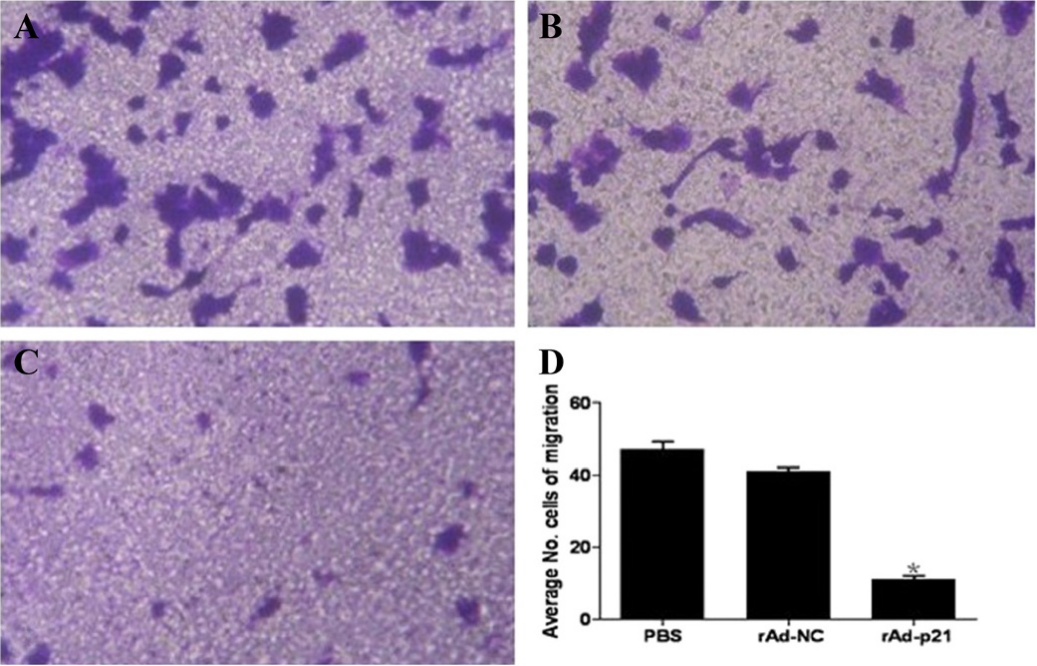
**Effect of the** ***p21*** **gene on the migration of cultured human RPE cells. A**: RPE PBS, **B** RPE rAd-NC, **C**: RPE rAd-p21, **D**: Bar graphs represent the number of migrating RPE cells in PBS, rAd-NC and rAd-p21 groups. Each value represents the mean ± SD of three independent experiments, each performed in triplicate (*P < 0.05).

To examine the effect of p21 on the migration of human RPE cells, Transwell assays were performed in the PBS, rAd-NC and rAd-p21 groups. As shown in Figure 2, rAd-p21 suppressed human RPE cell migration compared with the PBS and rAd-NC groups *in vitro*. This result demonstrated that overexpression of p21 inhibited the migration of human RPE cells.

### Overexpression of p21 inhibits levels of CDK2 and CyclinE *in vitro*and *in vivo*

**Figure 3:**
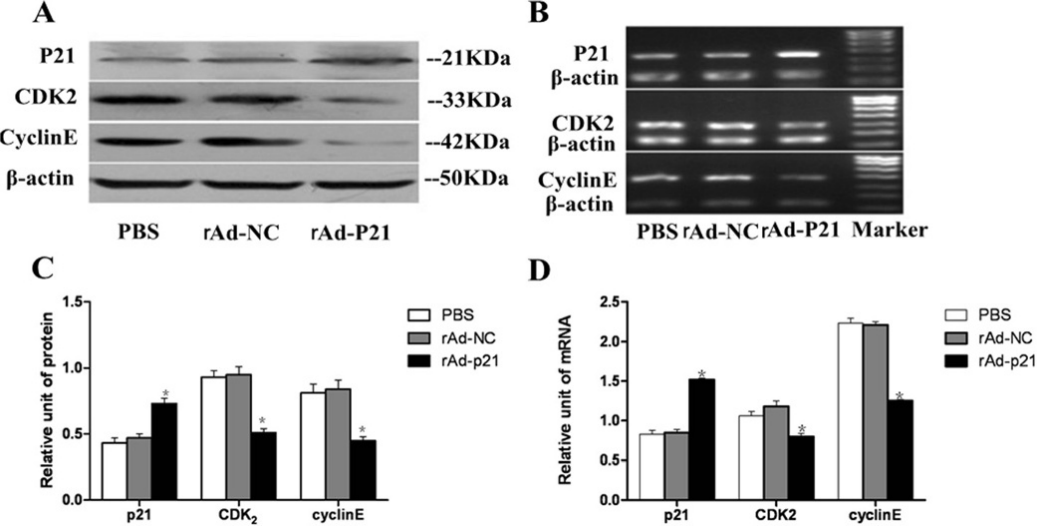
**Protein and mRNA levels of p21, CDK2 and cyclinE in human RPE cells was examined by western blotting and RT-PCR analysis, respectively. A**: Expressions of P21, CDK_2_ and cyclinE were assessed by western blot analysis in PBS, rAd-NC and rAd-p21 groups. β-Actin was used as the control. **B**: Expression of p21, CDK_2_ and cyclinE mRNA were assessed by RT-PCR analysis. **C**, **D**: Quantitative analysis of proteins and mRNA by densitometric scan. (n = 6). The error bars represent the standard deviation. There were statistically significant differences in p21, CDK_2_ and cyclinE protein and mRNA levels in the rAd-p21 group compared with the other two groups. (*p < 0.05).

To further confirm the function of p21, we transfected rAd-p21 into cultured human RPE cells, which showed that p21 expression in RPE cells was significantly increased at both protein and mRNA levels in the rAd-p21 group compared with the PBS and rAd-NC groups. Meanwhile, expression of CDK_2_ and cyclinE was depressed in the rAd-p21 group compared with the PBS and rAd-NC groups (Figure 3). These results suggested that overexpression of p21 suppressed the expression of CDK_2_ and cyclin E *in vitro*.

**Figure 4:**
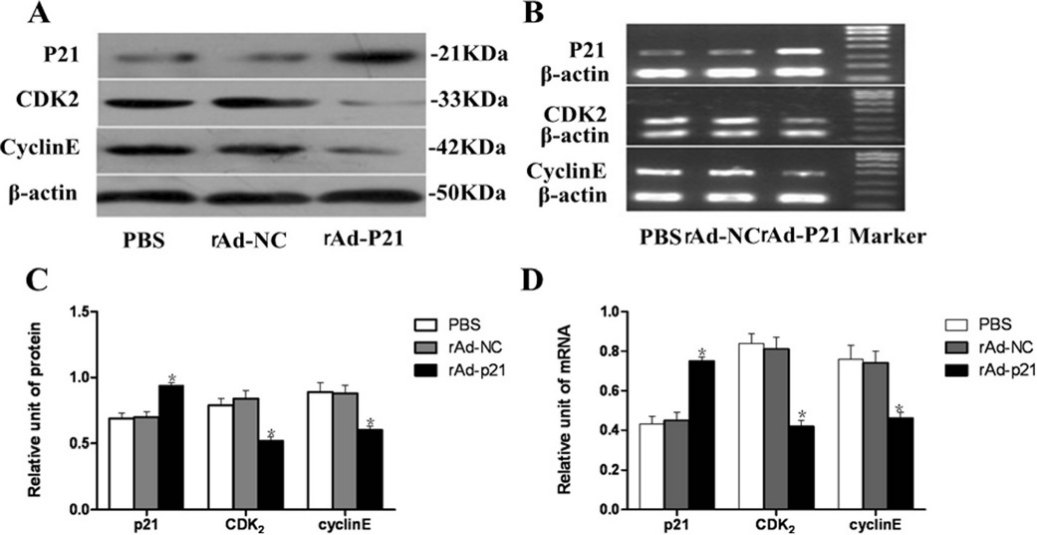
**Retinal expression of p21, CDK2 and cyclinE protein and mRNA levels after treatment analyzed by western blotting and RT-PCR assays, respectively. A**: Expression of P21, CDK_2_ and cyclinE were assessed by western blot analysis in PBS, rAd-NC and rAd-p21 groups *in vivo*. **B**: Expressions of p21, CDK_2_ and CyclinE mRNA were assessed by RT-PCR analysis in the three groups *in vivo.* **C**, **D**: Quantitative analysis of proteins and mRNA by densitometric scans (n = 6). The columns are the mean ± SD of three independent groups. There were statistically significant differences in p21, CDK_2_ and cyclinE protein and mRNA levels in the p21 gene treated group *in vivo* compared with the other two groups (*p < 0.05).

To investigate the potential role of the *p21* gene for cell cycle regulatory proteins in the inhibition of experimental PVR, we examined retinal protein and mRNA levels of p21, CDK_2_ and cyclinE after injecting rAd-p21 into the vitreous. Western blot and RT-PCR analysis of total retinal lysates indicated that p21 expression was significantly increased both at the protein and mRNA levels in the gene treated group compared with the PBS and empty vector groups, whereas expression of CDK_2_ and cyclinE were suppressed in the rAd-p21 treated group compared with the PBS and empty vector groups (Figure 4). The results were consistent with the experiment *in vitro*. This result showed that rAd-p21 could act on the retina and overexpress the protein p21.

### Effect of rAd-p21 on the inhibition of experimental PVR

**Figure 5:**
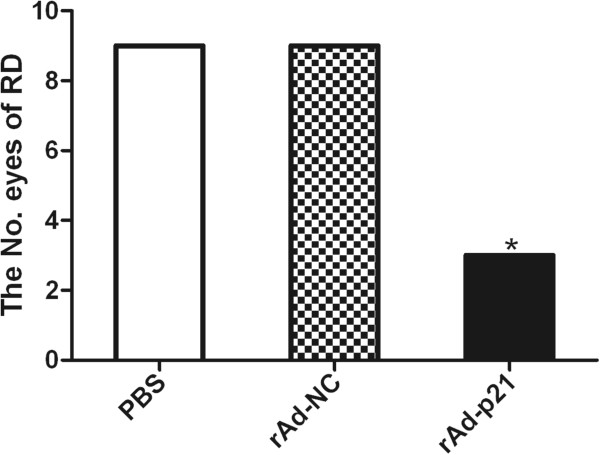
**Number of eyes with retinal detachment (RD).** There were statistically significant differences in the number of eyes with RD in the rAd-p21 group *in vivo* compared with the other two groups on the 21^st^ day after treatment (*p < 0.05).

**Figure 6:**
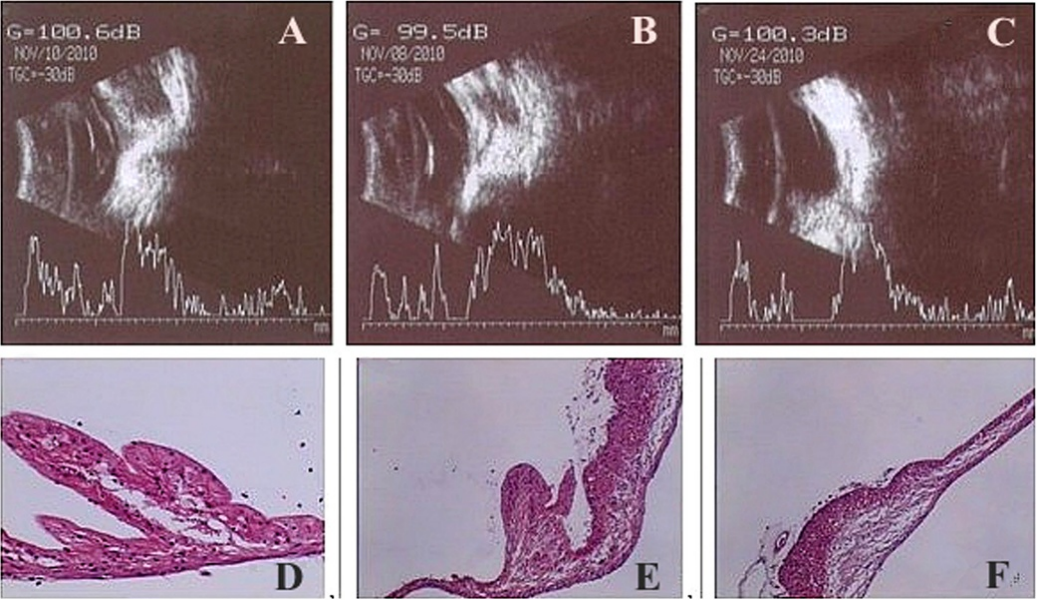
**B scans and H&E staining (B scan, A-C; H&E staining, D-F, 200×).** The retinas of rabbits at 21 days after treatment revealed that rAd-p21 significantly inhibited PVR. B scans showed significantly proliferative membranes with severe retinal detachment in the PBS and rAd-NC groups **(A,B)**. However, rAd-p21 treatment inhibited proliferative membranes without severe retinal detachment. **(C)** H&E staining indicated that retinal microanatomy had more integrity in the rAd-p21 group **(F)** than in groups PBS and rAd-NC **(D,E)**.

In all groups, we found that the stage of PVR progressed steadily over time by examination with indirect ophthalmoscopy. Proliferative membranes were formed without retinal detachment in the rabbit eyes in all groups on the 3^rd^ day after injection. There were massive proliferative membranes on the surface of the retina with focal retinal detachment in the empty vector and PBS groups on the 7^th^ day after injection; however, there were fewer proliferative membranes with or without retinal detachment in the gene treated group than the control groups. Then tractional retinal detachment developed in the posterior retina on the 14^th^ day and in the peripheral retina with total retinal detachment on the 21^st^ day in the control groups. However, eyes with retinal detachment in the gene treated group on the 14^th^ day were less prevalent than in the control group, and there was no total retinal detachment on the 21^st^ day. The stages of groups according to Fastenberg on the 21^st^ day after treatment are shown in Table 1. The eyes in stage 4 and stage 5 were thought to have retinal detachments. The results showed that there were statistically significant differences in the rAd-p21 group compared with the other two groups on the 21^st^ day after treatment (Figure 5). B scans and H&E staining of rabbit retinas on the 21^st^ day showed proliferative membranes with severe retinal detachment in the PBS and empty vector groups. However, intravitreal injection of rAd-p21 inhibited proliferative membranes without severe retinal detachment. This result indicated that overexpression of p21 inhibited PVR progression (Figure 6).

**Table 1:** **Stages of groups according to Fastenberg on the 21st day after treatment**

Stage	PBS group	rAd-NC group	rAd-p21 group
0	0	0	0
1	0	0	1
2	0	0	1
3	1	1	5
4	4	3	3
5	5	6	0

## Discussion

PVR is a vision-threatening disease and a common complication after surgery to correct rhegmatogenous retinal detachment. PVR is a proliferative response within the eye. Its pathological processes include inflammatory cell infiltration, proliferation of cell growth and the formation of scars. Other studies have demonstrated that fibrocellular membranes in PVR mainly consist of RPE cells, macrophages, glial cells, periostin and fibroblast-like cells [12, 13]. Injection of those cells into the vitreous in animal models, whether they are macrophages, fibroblasts or RPE cells, results in pathology that mimics PVR [14, 15]. During the course of PVR formation, the proliferation and migration of RPE cells is a very important step. RPE cells move from under the photoreceptor layer into the vitreous cavity and stay on the retinal surface to form an epiretinal membrane. However, some studies have already shown that RPE cells are very poor inducers of PVR alone when injected in a rabbit eye. Therefore, we chose to use RPE cells in an *in vitro* experiment and RPE cells and PRP to induce PVR in an animal model.

P21 is known as an important CDKI with a wide range of kinase inhibition activity, which can effectively inhibit CDK_2_ and cyclins to prevent cells from going through the G1/S phase checkpoint, and to inhibit cell proliferation [16, 17]. Previous studies have shown that p21 suppressed tumor cell migration and proliferation [9, 10]. PVR has many parallels to cancer. Antiproliferative and cell cycle blocking agents are commonly used in the treatment of PVR [18, 19]. Furthermore, p21 showed low expression levels in PRE-19 cells delivered from human RPE cells, which are important in the pathogenesis of PVR [20]. However, there have been no reports of p21 gene therapy in PVR. Finally, our study indicated that p21 has a role in the suppression of PRE cell proliferation and migration *in vitro*, and inhibits PVR *in vivo*.

Adenovirus-mediated gene transfection technology has been widely applied in research on ophthalmic diseases [21–23]. For example, clinical trials have indicated that adenovirus vectors containing wild type p53 was a safe, feasible gene-therapy approach [24]. In our study, p21 was successfully transfected into the human D407 RPE cell line by an adenovirus vector *in vitro*, and the protein and gene of p21 showed significantly higher expression in the rAd-p21 group compared with the rAd-NC group.

Up-regulation of p21 arrested the cell cycle at the G1 phase, and the number of cells in the G0 and G1 phases was significantly increased. P21 belongs to the Cip/Kip family, which is one of the CDKIs, and can block the activity of cyclin/CDK complexes, specifically CDK_2_ in E/A-CDK2 cyclin complexes [3]. These complexes block the cell-cycle transition from the G1 phase to the S phase. P21 is regulated by two different pathways, i.e., a p53-dependent pathway (DNA damage leading to the activation of p53 and upregulation of p21 causing cell cycle blockage in the G1 phase with possible DNA repair or induction of apoptosis), and a p53-independent pathway (through cell growth factors, such as interferon-β and tumor necrosis factor-α, which are able to induce p21 in p53-deficient cells in quiescence) [25, 26]. Our results also indicated that p21 overexpression was connected with the depression of CDK_2_ and cyclinE in human RPE cells, leading to arrest of the cell cycle and reduction of the proliferation of cells.

Cell migration is another important process in the development of PVR. Without migration, RPE cells would not be involved in the formation of a proliferative epiretinal membrane by entering the vitreous cavity and settling down on the surface of the retina. In this study, p21 overexpression inhibited the migration of RPE cells, which is consistent with its effects on the migration of vascular smooth muscle cells (SMCs), epithelial cells and cancer cells [27–30]. Previous studies have reported that the migration of RPE cells depended on the PI3K/AKT and MAPK signaling pathways, and p21 inducing cell cycle progression and survival also depended on PI3K/AKT and MAPK activations [31–33]. Therefore, the potential mechanism of p21 depressing RPE cell migration probably depends on PI3K/AKT and MAPK signaling pathways. The results will be verified by further experiments.

Experimental models of PVR were conducted to investigate intraocular proliferation. Intraocular injection of RPE cells and PRP to establish a PVR animal model is one of the commonly used methods that can induce intravitreal proliferative responses. The response will reach a peak for nearly 2 weeks and develop tractional retinal detachment at 3–4 weeks after injection [34]. Furthermore, we have previously shown that the expression of p21 decreased gradually from 7 days and reached the lowest point at 2 weeks after PVR induction. The expression of p21 and PVR development has a negative correlation. In agreement with previous studies, we administered rAd-p21 by intravitreal injection 7 days after PVR induction. Increased mRNA and protein expression of p21 *in vivo* by western blotting and RT-PCR indicated that rAd-p21 could incorporate within the retina. The development of a proliferative membrane and retinal detachment, shown by B scans and HE staining, in the gene treated group was lighter than in the control groups. Western blot and RT-PCR showed that the expression of p21 was up-regulated, whereas the expression of CDK_2_ and cyclinE were down-regulated after treatment. These results were consistent with the results *in vitro*. Our study indicated that treatment with p21 not only inhibited human RPE cell proliferation and migration *in vitro* but also probably arrested the RPE cells in the G1 phase by regulating CDK_2_ and cyclinE activity to inhibit the formation of proliferative membranes and slowed down PVR development procession in the rabbit PVR model. The signal pathway of p21 inhibited PVR is still not clear. Previous studies reported that the regulation of p21 was controlled at the transcriptional level by both p53 dependent and independent mechanisms [35]. The relationship between p21 and p53 was mainly studied in tumors, and is perhaps similar in PVR.

During the treatment *in vivo*, all rabbits tolerated the treatment well and there was no increase in systemic toxicity. In addition, there was no cataract, glaucoma, or endophthalmitis found by slit lamp and indirect ophthalmoscopy during the treatment.

As is known, although RPE cells are main cells that participate in epiretinal membrane formation, macrophages and fibroblasts also play key roles in this progression. Further research is necessary to determine whether increased expression of p21 suppresses PVR in those cells.

## Conclusions

Our results suggested a role for the p21 gene in the inhibition of the proliferation and migration of PRE cells *in vitro*, and showed the *p21* gene can reduce the occurrence of severe PVR cases through inhibition the expression of CDK_2_ and cyclin E. P21 gene may be a new therapeutic target for PVR.
